# High frequency of diabetic ketoacidosis at diagnosis of type 1 diabetes in Italian children: a nationwide longitudinal study, 2004–2013

**DOI:** 10.1038/srep38844

**Published:** 2016-12-19

**Authors:** Valentino Cherubini, Edlira Skrami, Lucia Ferrito, Stefano Zucchini, Andrea Scaramuzza, Riccardo Bonfanti, Pietro Buono, Francesca Cardella, Vittoria Cauvin, Giovanni Chiari, Giuseppe d′Annunzio, Anna Paola Frongia, Dario Iafusco, Ippolita Patrizia Patera, Sonia Toni, Stefano Tumini, Ivana Rabbone, Fortunato Lombardo, Flavia Carle, Rosaria Gesuita, Diabetes Study Group of the Italian Society for Pediatric Endocrinology and Diabetology (ISPED), Riccardo Lera, Livia De Luna, Antonella Gualtieri, Clara Zecchino, Elvira Piccinno, Petra Reinstadler, Elena Prandi, Francesco Gallo, Gianfranco Morganti, Carlo Ripoli, Alfonso La Loggia, Piera Scanu, Giuliana Cardinale, Letizia Grazia Tomaselli, Felice Citriniti, Nicola Lazzaro, Valeria De Donno, Benedetta Mainetti, Maria Susanna Coccioli, Rosella Maccioni, Ugo Marongiu, Mariella Bruzzese, Antonio Iannilli, Daniela Pardi, Santino Confetto, Angela Zanfardino, Lorenzo Iughetti, Adriana Franzese, Francesco Cadario, Anna Franca Milia, Gavina Piredda, Miriam Soro, Antonella Correddu, Alfonso Galderisi, Fiorella De Berardinis, Giovanni Federico, Giorgio Zanette, Tosca Suprani, Annalisa Pedini, Maria Luisa Manca Bitti, Maurizio Delvecchio, Michela Trada, Gianfranco Meloni, Alberto Gaiero, Pasquale Bulciolu, Lucia Guerraggio, Elena Faleschini, Manuela Zanatta, Alessandro Salvatoni, Claudio Maffeis, Claudia Arnaldi

**Affiliations:** 1 Division of Paediatric Diabetes, Women’s and Children’s Health, AOU Ancona, Salesi Hospital, Ancona, Italy; 2 Centre of Epidemiology and Biostatistics, Polytechnic University of Marche, Ancona, Italy; 3 Department of Pediatrics, S. Orsola-Malpighi Hospital, Via Albertoni 15, 40138 Bologna, Italy; 4 Department of Pediatrics, Azienda Ospedaliera, “Ospedale Luigi Sacco”, University of Milan, Via G.B. Grassi 74, 20157 Milan, Italy; 5 Department of Pediatrics, Endocrine Unit, Scientific Institute Hospital San Raffaele, Vita-Salute University, Via Olgettina 60, 20132 Milan, Italy; 6 UOSD Pediatric Diabetology, ASL NA2 Nord, Via Corrado Alvaro 8, Monteruscello, 80072 Pozzuoli, Italy; 7 Department of Pediatrics, U.O.S. Pediatric Diabetology, ARNAS Civico Di Cristina, Via Benedettini 1, 90134 Palermo, Italy; 8 Pediatric Unit, S. Chiara Hospital, Largo Medaglie d’Oro 9, 38122 Trento, Italy; 9 Postgraduate School of Pediatrics, University of Parma, Viale Gramsci 14, 43100 Parma, Italy; 10 Giannina Gaslini Institute, Via Gaslini 5, 16147 Genova, Italy; 11 Unit of Pediatric Diabetes, Brotzu Hospital, Piazzale Ricchi 1, 09134 Cagliari, Italy; 12 Department of Pediatrics, Second University of Naples, Via S. Andrea delle Dame 4, 80138 Naples, Italy; 13 Endocrinology and Diabetes Unit, University Department of Pediatric Medicine, Bambino Gesù Children’s Hospital, Piazza Sant’Onofrio 4, 00165 Rome, Italy; 14 Juvenile Diabetes Center, Anna Meyer Children’s Hospital, Via Pieraccini 24, 50132 Florence, Italy; 15 Center of Pediatric Diabetology, University of Chieti, 66100 Chieti, Italy; 16 Department of Pediatrics, University of Turin, Piazza Polonia 94, 10126 Turin, Italy; 17 Department of Pediatrics, Alessandria Hospital, Alessandria, Italy; 18 Department of Pediatrics, Alghero Hospital, Alghero, Italy; 19 Department of Pediatrics, Avezzano Hospital, Avezzano, Italy; 20 Department of Pediatrics, Bari Hospital, Bari, Italy; 21 Department of Pediatrics, Bolzano Hospital, Bolzano, Italy; 22 Department of Pediatrics, Brescia Hospital, Brescia, Italy; 23 Department of Pediatrics, Brindisi Hospital, Brindisi, Italy; 24 Department of Pediatrics, Busto Arsizio Hospital, Busto Arsizio, Italy; 25 Department of Pediatrics, Pediatric Clinic Hospital, Cagliari, Italy; 26 Department of Pediatrics, Caltanissetta Hospital, Caltanissetta, Italy; 27 Department of Pediatrics, Carbonia Hospital, Carbonia, Italy; 28 Department of Pediatrics, Casarano Hospital, Casarano, Italy; 29 Department of Pediatrics, Catania Hospital, Catania, Italy; 30 Department of Pediatrics, Catanzaro Hospital, Catanzaro, Italy; 31 Department of Pediatrics, Crotone Hospital, Crotone, Italy; 32 Department of Pediatrics, Cuneo Hospital, Cuneo, Italy; 33 Department of Pediatrics, Forlì Hospital, Forlì, Italy; 34 Department of Pediatrics, Francavilla Fontana Hospital, Francavilla Fontana, Italy; 35 Department of Pediatrics, Iglesias Hospital, Iglesias, Italy; 36 Department of Pediatrics, Lanusei Hospital, Lanusei, Italy; 37 Department of Pediatrics, Locri Hospital, Locri, Italy; 38 Department of Pediatrics, Massa Carrara Hospital, Massa Carrara, Italy; 39 Department of Pediatrics, Messina Hospital, Messina, Italy; 40 Department of Pediatrics, Modena Hospital, Modena, Italy; 41 Department of Traslational Sciences, University Federico II, Naples, Italy; 42 Department of Pediatrics, Novara Hospital, Novara, Italy; 43 Department of Pediatrics, Nuoro Hospital, Nuoro, Italy; 44 Department of Pediatrics, Olbia Hospital, Olbia, Italy; 45 Department of Pediatrics, Oristano Hospital, Oristano, Italy; 46 Department of Pediatrics, Ozieri Hospital, Ozieri, Italy; 47 Department of woman’s and Child’s health, University of Padova, Padova, Italy; 48 Department of Pediatrics, Paola Hospital, Paola, Italy; 49 Department of Pediatrics, Pisa Hospital, Pisa, Italy; 50 Department of Pediatrics, Pordenone Hospital, Pordenone, Italy; 51 Department of Pediatrics, Ravenna Hospital, Ravenna, Italy; 52 Department of Pediatrics, Rimini Hospital, Rimini, Italy; 53 Department of Pediatrics, Tor-Verg Hospital, Rome, Italy; 54 Department of Pediatrics, Casa Sollievo della Sofferenza Hospital, San Giovanni Rotondo, Italy; 55 Department of Pediatrics, Sanremo Hospital, Sanremo, Italy; 56 Department of Pediatrics, Sassari Hospital, Sassari, Italy; 57 Department of Pediatrics, Savona Hospital, Savona, Italy; 58 Department of Pediatrics, Tempio Pausania Hospital, Tempio Pausania, Italy; 59 Department of Pediatrics, Tradate Hospital, Tradate, Italy; 60 Department of Pediatrics, Trieste Hospital, Trieste, Italy; 61 Department of Pediatrics, Udine Hospital, Udine, Italy; 62 DSCM-Pediatric Unit, University of Insubria, Varese, Italy; 63 Department of Pediatrics, Verona Hospital, Verona, Italy; 64 Department of Pediatrics, Viterbo Hospital, Viterbo, Italy

## Abstract

This longitudinal population-based study analyses the frequency of diabetic ketoacidosis (DKA) at type 1 diabetes diagnosis in Italian children under 15 years of age, during 2004–2013. DKA was defined as absent (pH ≥ 7.30), mild/moderate (7.1 ≤ pH < 7.30) and severe (pH < 7.1). Two multiple logistic regression models were used to evaluate the time trend of DKA frequency considered as present versus absent and severe versus absent, adjusted for gender, age group and geographical area of residence at diagnosis. Overall, 9,040 cases were ascertained. DKA frequency was 40.3% (95%CI: 39.3–41.4%), with 29.1% and 11.2% for mild/moderate and severe DKA, respectively. Severe DKA increased significantly during the period (OR = 1.03, 95%CI: 1.003–1.05). Younger-age children and children living in Southern Italy compared to Central Italy were at significantly higher risk of DKA and severe DKA. Family history of type 1 diabetes and residence in Sardinia compared to Central Italy were significantly associated with a lower probability of DKA and severe DKA. The high frequency of ketoacidosis in Italy over time and high variability among age groups and geographical area of residence, strongly suggests a continuing need for nationwide healthcare strategies to increase awareness of early detection of diabetes.

Diabetology Despite increasing social awareness of diabetes^1^, diabetic ketoacidosis (DKA) still remains a major cause of diabetes-related morbidity and mortality in young people^2^. Although the frequency of DKA varies widely between countries, levels of DKA at diagnosis of type 1 diabetes seem to be constant in Germany^3^, Great Britain^4^, Austria^5^ and the Unites States^6^. A recent Italian study on DKA incidence at diagnosis in Italy during the calendar years 2012–2013 showed a higher frequency of DKA in subjects under 18 years of age than that observed in Northern European countries^7^. The Parma campaign demonstrated the effectiveness of a public education programme in reducing the frequency of DKA at diabetes diagnosis in a restricted area. A subsequent analysis suggested such prevention campaigns should be repeated periodically in order to maintain effectiveness over time^8^. As a result of the positive effect of the Parma campaign, local prevention programmes spread spontaneously throughout Italy. Moreover, the provision of free primary care services for children under the national health system of Italy facilitates a more focused intervention programme via family paediatricians. Of interest, it has recently been reported that a family history of type 1 diabetes and a higher background incidence of type 1 diabetes were associated with a lower risk of DKA at diabetes diagnosis^9^.

The aim of this longitudinal population-based study was to analyse the frequency of DKA at the time of diagnosis of type 1 diabetes over a 10-year period in Italian children. Although type 1 diabetes has been increasing in recent decades, we can hypothesize that DKA frequency at diabetes diagnosis should be stable or decreasing due to the effects of prevention programs and increasing social awareness.

## Research Design and Methods

### Data source

The Study Group for Diabetes (SGD) of the Italian Society of Pediatric Endocrinology and Diabetes (ISPED), which includes all the Italian centres for diabetes in children and adolescents^10^, promoted a nationwide longitudinal population-based study on DKA at diagnosis of type 1 diabetes.

The same methodology to collect and record clinical data was used by all centres. This methodology, which was already widely used by the centres, became a systematic clinical practice when the Italian Insulin-Dependent Diabetes Registry (RIDI)^11^ was established in 1997.

All children under 15 years of age with a new diagnosis of type 1 diabetes, according to International Society for Pediatric and Adolescent Diabetes (ISPAD) criteria^12^, were included in the study. The principal investigators of each participating local centre were asked to verify the diagnosis of type 1 diabetes in each new case in order to exclude all other forms of diabetes.

### Variables

Date of birth and of diabetes diagnosis, gender, municipality and province of residence at diagnosis, first-degree relative(s) with type 1 diabetes, venous pH, bicarbonate levels, information on cerebral oedema, permanent neurological dysfunctions (motor deficits, visual impairments, memory loss, speech problems such as dysphasia and dysarthria) and death were obtained for all incident diabetes cases presenting between 1 Jan 2004 and 31 Dec 2013. Cerebral oedema was defined according to ISPAD criteria^13^. Oedema and death occurring within 30 days of diagnosis were considered to be related to the diagnosis of diabetes. Permanent neurological dysfunctions were verified one year after diagnosis of diabetes, in order to exclude transient neurological dysfunctions. The participating centres prospectively recorded all the new cases of diabetes presenting in each year of the study period; therefore, the completeness of the data collected concerning diabetes cases was close to 100%.

The data were gathered between 1 January and 31 May 2014 by the study coordinating centre, using a standardized data format. This centre was responsible for data quality control, in order to minimize missing values and pH values, in particular, since pH is the variable used to define DKA. If cases of incomplete data were identified, the participating centres were asked to verify the data and provide any missing information. Venous pH was reported for all the cases, while bicarbonate values were reported in about 60% of cases. Moreover, when the municipality of residence at diagnosis was missing, the province of residence was used to identify the geographical macro-area to which to assign cases.

ISPAD criteria^13^ were used to define DKA: absent (pH ≥ 7.30), mild (7.2 ≤ pH < 7.30), moderate (7.1 ≤ pH < 7.20) and severe (pH < 7.1). As the clinical implications for mild and moderate DKA are similar, they were considered together. Residence at diagnosis was defined by main Italian geographical macro-area: North, Centre, South (including Sicily) and Sardinia. Age at diagnosis was grouped in three classes (0–4, 5–9, and 10–14 years).

All procedures were conducted in accordance with the ethical standards of the responsible committee. Written or verbal informed consent was not required, as this study was based on data routinely recorded in clinical practice.

### Statistical methods

The frequency of DKA at type 1 diabetes diagnosis was evaluated as punctual and 95% confidence interval (95% CI) estimates using the binomial distribution, and analysed according to age group, gender, geographical area of residence at diagnosis, family history of type 1 diabetes and year of diagnosis. Comparisons between groups were performed by the chi-square test or by Fisher’s exact test when expected frequencies were lower than five.

Two multiple logistic regression models were used to evaluate the time trend of DKA frequency. DKA at diagnosis was the dependent variable, considered as present versus absent in the first model and as severe versus absent in the second. Year of diagnosis, gender, age class, and geographic area of residence (Central Italy was the reference category) were used as independent variables. The Likelihood Ratio (LR) test was used to detect the variables to be included in each model and the models’ goodness of fit was evaluated by the Hosmer and Lemeshow test. All estimates were evaluated using the 95% CI.

All the statistical analyses were performed using the R.3.1.2 statistical package; a probability lower than 0.05 was used to assess statistical significance.

## Results

In total, the 59 centres ascertained a total of 9,040 cases. The participating centres and the number of cases for each centre can be found in the supplementary data (Supplementary Table 1).

Overall DKA frequency was 40.3% (95% CI: 39.3–41.4%). During the study period, mild/moderate ketoacidosis at diabetes diagnosis occurred in 2,633 children, 29.1% (95% CI: 28.2–30.1%), and severe DKA in 1013 children, 11.2% (95% CI: 10.6–11.9%).

Table 1 shows the distribution of DKA according to the characteristics of the children. A significantly higher frequency of DKA was found in children under 5 years of age and in those living in Southern Italy at diabetes diagnosis. The frequency of DKA was found to be significantly lower in children living in Sardinia or with at least one first-degree relative with type 1 diabetes.

**Figure 1:**
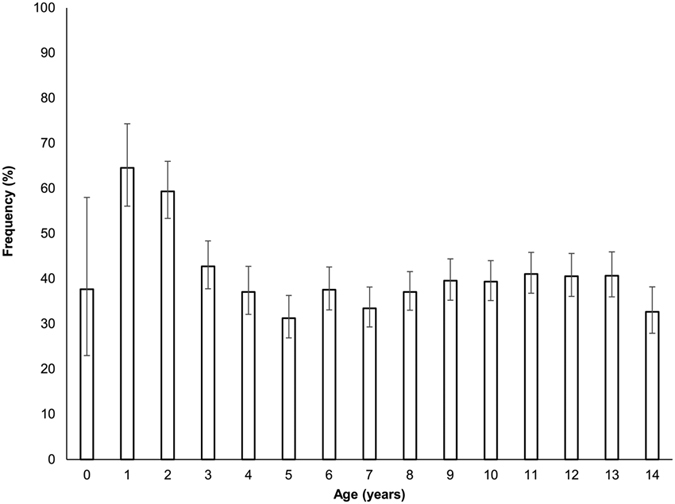
Frequency of diabetic ketoacidosis and 95% confidence intervals according to age at type 1 diabetes diagnosis.

The highest frequencies of DKA at diabetes diagnosis were found in children of 1 and 2 years of age, as shown in Fig. 1.

Table 2 shows the DKA time trend estimates, adjusted for covariates, that resulted from the two multiple logistic regression analyses. There was a significant trend toward an increase in the incidence of severe DKA over time [Odds ratio (OR) = 1.03, 95% CI: 1.003–1.05, p = 0.028], with an average increase of about 3% per year during the study period. No significant trend was found in overall incidence of DKA (OR = 1.02, 95% CI: 1.00–1.03, p = 0.060). Children 0–4 years old were found to be at significantly higher risk of DKA, overall as well as severe DKA, compared with the other two age groups (5–9 and 10–14 years).

Children in the 0–4 age group were about twice as likely to have DKA and severe DKA than those 5–9 years old (OR = 1.85, 95% CI: 1.65–2.07, p < 0.001 and OR = 2.09, 95% CI: 1.75–2.49, p < 0.001 for DKA and severe DKA, respectively) and at about 60% higher risk of DKA and 75% higher risk of severe DKA than children aged 10–14 years (OR = 1.60, 95% CI: 1.43–1.79, p < 0.001, OR = 1.75, 95% CI: 1.47–2.08, p < 0.001, respectively for DKA and severe DKA).

A family history of type 1 diabetes was significantly associated with DKA: the probability of DKA and of severe DKA decreased by about 70% in children having at least one first-degree relative with type 1 diabetes, compared with children without a family history of type 1 diabetes (OR = 0.32, 95% CI: 0.26–0.40, p < 0.001 and OR = 0.29, 95% CI: 0.19–0.42, p < 0.001, respectively for DKA and severe DKA). Geographical area of residence was associated with DKA: children living in the Southern Italy were found to be at significantly higher risk of DKA and severe DKA compared with children living in Central Italy (OR = 2.19, 95% CI: 1.92–2.49, p < 0.001, and OR = 1.85, 95% CI: 1.51–2.28, p < 0.001, respectively for DKA and severe DKA). Children living in Sardinia were found to be at significant lower risk of DKA and severe DKA compared with children living in the Central Italy (OR = 0.69, 95% CI: 0.56–0.85, p < 0.001 and OR = 0.68, 95% CI: 0.48–0.95, p = 0.026, respectively for DKA and severe DKA).

### Cerebral oedema, permanent neurological dysfunctions and death

Cerebral oedema occurred in 109 newly-diagnosed cases of type 1 diabetes (1.3%, 95% CI: 1.0–1.5). 105 of these cases of cerebral oedema occurred in children with DKA (3%, 95% CI: 2.4–3.6); of these 24 were children with mild/moderate DKA (0.9%, 95% CI: 0.6–1.4) and 81 were children with severe DKA (8.2%, 95% CI: 6.6–10.1). Most of the cases were females (60.6%) living in Southern (33.9%) and Central Italy (31.2%); 3.7% of cases had at least a first-degree relative with type 1 diabetes. The mean age was 7 years [standard deviation (SD) = 3.9 years].

Permanent neurological dysfunction occurred in 7 cases of new diagnosis of diabetes (0.08%, 95% CI: 0.03–0.17); the mean age was 4 years (SD = 3 years); 3 of these cases occurred in children living in Southern Italy and 4 in children living in Central Italy; 6 were males; 6 of the 7 had severe DKA and 5 of the 7 developed cerebral oedema.

During the study period, four deaths related to type 1 diabetes were recorded (0.04%, 95% CI: 0.01–0.11). In two cases the subjects had severe DKA, were living in Northern Italy and in Sardinia and were 2 years old; one had mild DKA, was living in Southern Italy and was 4 years old; one had no DKA, was living in Northern Italy and was 5 years old. The deaths occurred within 2–4 days of diagnosis of type 1 diabetes in the cases from Northern Italy (n = 2) and Sardinia (n = 1) and within 29 days in the case from Southern Italy. None of the cases had a family history of type 1 diabetes.

## Discussion

This is the first study based on a large number of new cases of type 1 diabetes collected over a long period of time and covering almost the entirety of the Italian population under 15 years of age. In mainland Italy, DKA frequency at time of diabetes diagnosis (41.9%) was consistent with recent reports from other countries, such as France (43.9%)^14^ and Brazil (42.3%)^15^, and with a recent Italian study based on two years of observation (38.5%)^7^. The DKA frequency in Italian children at diagnosis of type 1 diabetes was somewhat higher than that reported in Austria (34.0%)^5^, Germany (21.1%)^3^, New Zealand (25.0%)^16^ and the U.S. (31.1%)^17^ and, disappointingly, close to twice that reported in Finland (22.4%)^18^ and Denmark (17.9%)^19^ and in Canada (18.6%)^20^. However, it should be noted that there were minor differences in the definition of DKA used in the studies.

On the other hand, the DKA frequency in Sardinia (23.6%) was lower than in mainland Italy and comparable with the frequencies reported in countries with a high incidence of type 1 diabetes, such as Finland^18^, where a negative relationship between the incidence of type 1 diabetes and the frequency of DKA at diabetes diagnosis was reported^1^,^21^. In countries with a high incidence of type 1 diabetes there is a correspondingly high level of overall awareness of the disease and the time between the first symptoms of diabetes and diagnosis is presumably reduced. This relationship could explain the huge difference between DKA frequency in Sardinia and in mainland Italy.

Moreover, a family history of type 1 diabetes is a preventive factor for presenting with DKA, due to increased awareness within families with previous experiences of diabetes and to the increased alertness of family physicians. We may therefore conclude that awareness of the disease, at both public and family levels, can have a positive effect in terms of correct and early diagnosis.

Diagnosis of type 1 diabetes is frequently associated with DKA in the younger age groups. This is consistent with our study, which reported that one out of two children under 5 years old and 60.7% under 2 years of age had DKA at diabetes diagnosis. Since DKA is both potentially life-threatening and preventable, implementation of a global health action plan involving physicians and the general population and targeted at very young children is strongly recommended.

Data of temporal trends in DKA at new diagnosis of type 1 diabetes over an extended period of time and in large populations of subjects under 15 years of age are limited. The overall frequency of DKA was found to be stable over time in a study of 14,664 youths in Germany and Austria in the period 1995–2007 (21.1%)^3^ as well as in another study of 5,615 children in the U.S. in the period 2002–2010 (ranging between 29.1% and 31.1%)^17^. These studies found that the frequency of DKA did not change over time, even following prevention campaigns, such as those conducted in Austria in 1989–2011^5^ and in Wales in 1999–2001^22^. In a Finnish population-based study in 585 children, a relative reduction of DKA, particularly among children <5 years of age, was observed in the period 1981–2001^21^. It was therefore unexpected to find an increasing frequency of severe DKA over time in our study.

This increasing frequency of severe DKA could be explained in part by the significantly increased incidence of type 1 diabetes in children aged <15 years in Southern Italy and Sardinia during 2004–2013. In contrast, it was substantially stable in Northern and Central Italy during this period^23^.

The high frequency of DKA cannot be related to the proximity of hospitals or different types of health care, since in all Italian regions there are one or more regional centres for paediatric diabetes, that can easily be reached by the population in under two hours^10^. Moreover, in all of the Italian centres, ISPAD recommendations on DKA are followed in clinical practice^13^.

In order to reduce the frequency of DKA at diabetes diagnosis, prompt diagnosis is crucial. This will depend on the family’s awareness of the first symptoms of diabetes and on the ability of the physician. A report from Ontario^20^ showed that children with diabetes presenting with DKA had a medical encounter before diagnosis more frequently than did those without DKA; this fact indicates a possible missed diagnosis.

In Italy, a regional network of family paediatricians, delivering free primary care to children under 15 years of age, has been operating since 1978. The territorial coverage reaches about 95% of children under 6 years of age and about 60% in the 6–14 years age group. However, confirmation of diagnosis may still be delayed by the process of referral for the necessary investigations, laboratory testing, and follow-up after the initial suspicion of diabetes, and a simple blood glucose stick test or immediate referral to a paediatric emergency room may be preferable whenever diabetes is suspected. Diagnosis may also be delayed when the generally non-specific early symptoms are initially mistaken for another disease. Acute patient management guidelines addressed to family paediatricians, implementation of clinical audits and DKA awareness campaigns may aid in early detection of the disease and avoid misdiagnosis.

The Parma campaigns implemented in 1991–1997 and in 1999–2006 were successful school- and physician-focused prevention campaigns that demonstrated the effectiveness of recognising the early warning symptoms of diabetes in reducing the risk of DKA^8^. The campaigns targeted children between 6 and 14 years of age attending schools in the Province of Parma, a very small area in Italy with a limited at-risk population of about 26,000 resident children, making use of posters displaying diabetes alert messages and practical guidance for parents. Nowadays, with the widespread use of social networking, it may be practical to organise awareness campaigns that reach the entire Italian school-age population (more than 8 million children).

Preventing DKA would not only limit hospitalizations, but would contribute to eliminating the most common cause of diabetes-related mortality and morbidity, including permanent DKA-related neurological dysfunction. Although our study found that mortality rates due to DKA were lower (0.04%) than those observed previously in developed countries like Canada, the U.S. and the United Kingdom, where they varied from 0.15% to 0.35%^24^, the continued occurrence of such outcomes in developed countries with well-developed health care systems should be considered unacceptable. For instance, the Italian health care system covers the whole resident population, is free of charge, and guarantees primary care for children through an extensive network of family paediatricians.

There were some limitations to this study. Even though paediatric diabetes centres are established by law and are distributed uniformly throughout Italy, we cannot exclude that children with new diagnoses of diabetes may be referred to diabetes centres for adults in some small areas of the country, where it is less easy to reach the care centres. Consequently, a small number of cases may have escaped inclusion in the study.

After the Parma campaign, many other similar initiatives were taken at the local level by other paediatric diabetes centres. Therefore, it is possible that the geographical variability found in our study may be explained in part by the geographical variability of those campaigns. However, we were unable to obtain data to confirm this. On the other hand, the effect and the impact of a prevention campaign on deaths and neurological dysfunctions may be effectively evaluated only where implemented in a large population over a long period of time and using standardised and validated methodology, which is a strength of our study. Furthermore, we hypothesize that the socio-economic status of parents could be a factor in explaining DKA incidence at diabetes diagnosis, but such information is not routinely collected and was therefore not available in the clinical records. Nevertheless, we are confident that our results are representative of the Italian population as a whole because the study was promoted by ISPED and every effort was made to ensure the participation of all the paediatric centres for diabetes care.

In conclusion, ketoacidosis at diagnosis of type 1 diabetes was found in 40.3% of Italian children, with a large variation in age groups and geographical areas of residence. The high frequency of DKA over time strongly suggests the need for a national-level coordinated strategy to adjust potential geographical differences in the health care system. Moreover, preventive measures need to be implemented in order to ensure early diagnosis and to avoid adverse outcomes. These measures may include prevention campaigns to disseminate specific guidelines for the management of the acute patient addressed to family paediatricians and to increase the awareness of DKA among the general population.

## Additional Information

**How to cite this article**: Cherubini, V. *et al*. High frequency of diabetic ketoacidosis at diagnosis of type 1 diabetes in Italian children: a nationwide longitudinal study, 2004–2013. *Sci. Rep.* **6**, 38844; doi: 10.1038/srep38844 (2016).

**Publisher's note:** Springer Nature remains neutral with regard to jurisdictional claims in published maps and institutional affiliations.

## Supplementary Material

Supplementary Table 1

## Figures and Tables

**Table 1:** Frequency of diabetic ketoacidosis at type 1 diabetes diagnosis according to the main characteristics of the children.

	pH ≥ 7.3 (n = 5394)		pH < 7.3 (n = 3646)		p
	n	% (95% CI)	n	% (95% CI)	
**Age group**					
0–4	1066	50.9 (48.8–53.1)	1030	49.1 (46.9–51.2)	<0.001
5–9	2231	63.9 (62.3–65.5)	1258	36.1 (34.5–37.7)	
10–14	2095	60.7 (59.0–62.3)	1358	39.3 (37.7–41)	
**Gender**					
F	2498	59.0 (57.5–60.5)	1738	41.0 (39.5–42.5)	0.215
M	2895	60.3 (58.9–61.7)	1908	39.7 (38.3–41.1)	
**Geographic area of residence**					
Northern Italy	2172	64.1 (62.4–65.7)	1219	35.9 (34.3–37.6)	<0.001
Central Italy	1001	66.5 (64.0–68.9)	505	33.5 (31.1–36.0)	
Southern Italy	1633	48.4 (46.8–50.2)	1740	51.6 (49.8–53.2)	
Sardinia	588	76.4 (73.2–79.3)	182	23.6 (20.7–26.8)	
**Family history of type 1 diabetes**					
No	4772	57.7 (56.6–58.7)	3502	42.3 (41.2–43.4)	<0.001
Yes	488	81.6 (78.3–84.7)	110	18.4 (15.3–21.7)	

p values refer to Chi square test.

**Table 2:** Effect estimate of year of diagnosis, age, gender, family history of type 1 diabetes (T1D) and residence on risk of diabetic ketoacidosis at diagnosis of T1D.

Variables	pH < 7.3 vs pH ≥ 7.3			pH < 7.1 vs pH ≥ 7.3		
	OR	95% CI	p	OR	95% CI	p
Year of diagnosis	1.02	1.00–1.03	0.060	1.03	1.003–1.05	0.028
Age group (0–4 vs 5–9 years)	1.85	1.65–2.07	<0.001	2.09	1.75–2.49	<0.001
Age groups (0–4 vs 10–14 years)	1.60	1.43–1.79	<0.001	1.75	1.47–2.08	<0.001
Gender (Male vs Female)	0.96	0.88–1.04	0.305	0.87	0.76–0.998	0.047
Familiarity for T1DM (Yes vs No)	0.32	0.26–0.40	<0.001	0.29	0.19–0.42	<0.001
Residence area (North vs Centre)	1.12	0.99–1.28	0.082	1.07	0.87–1.32	0.534
Residence area (South/Sicily vs Centre)	2.19	1.92–2.49	<0.001	1.85	1.51–2.28	<0.001
Residence area (Sardinia vs Centre)	0.69	0.56–0.85	<0.001	0.68	0.48–0.95	0.026

Results of the multiple logistic regression analyses. Odds ratios (ORs) for the age groups are calculated by reversing the OR, with age group 0–4 years as reference category. First model (pH < 7.3 vs pH ≥ 7.3): Hosmer and Lemeshow test:χ^2^ = 9.775, df = 8, p-value 0.281; LR test: χ^2^ = 546, df = 8, p-value < 0.001. Second model (pH < 7.1 vs pH ≥ 7.3): Hosmer and Lemeshow test: χ^2^ = 8.411, df = 8, p-value 0.394; LR test: χ^2^ = 212, df = 8, p-value < 0.001.
