# Supplementary material for: High frequency of diabetic ketoacidosis at diagnosis of type 1 diabetes in Italian children: a nationwide longitudinal study, 2004–2013
Source: Sci Rep. 2016 Dec 19;6:38844. doi: 10.1038/srep38844 (PMC5171855; doi:10.1038/srep38844)
Supplement: Supplementary Table 1 [file srep38844-s1.doc]

SUPPLEMENTARY INFORMATION

**High frequency of diabetic ketoacidosis at diagnosis of type 1 diabetes in Italian children: a nationwide longitudinal study, 2004-2013**

Valentino Cherubini, Edlira Skrami, Lucia Ferrito, Stefano Zucchini, Andrea Scaramuzza, Riccardo Bonfanti, Pietro Buono, Francesca Cardella, Vittoria Cauvin, Giovanni Chiari, Giuseppe d’Annunzio, Anna Paola Frongia, Dario Iafusco, Ippolita Patrizia Patera, Sonia Toni, Stefano Tumini, Ivana Rabbone, Flavia Carle, Rosaria Gesuita and Diabetes Study Group of the Italian Society for Pediatric Endocrinology and Diabetology (ISPED)

**Supplementary Table 1.** List of centres participating to the study and the respective number of type 1 diabetes cases

| **Centre** | **N** | **%** |  | **Centre** | **N** | **%** |
| --- | --- | --- | --- | --- | --- | --- |
| Alessandria | 57 | 0.6 |  | Naples - Sun | 1030 | 11.4 |
| Alghero | 16 | 0.2 |  | Novara | 124 | 1.4 |
| Avezzano | 14 | 0.2 |  | Nuoro | 48 | 0.5 |
| Bari | 419 | 4.6 |  | Olbia | 93 | 1.0 |
| Bologna | 163 | 1.8 |  | Oristano | 37 | 0.4 |
| Bolzano | 151 | 1.7 |  | Padova | 204 | 2.3 |
| Brescia | 273 | 3.0 |  | Palermo | 467 | 5.2 |
| Brindisi | 48 | 0.5 |  | Paola | 21 | 0.2 |
| Busto Arsizio | 18 | 0.2 |  | Parma | 178 | 2.0 |
| Cagliari | 254 | 2.8 |  | Pisa | 70 | 0.8 |
| Caltanissetta | 102 | 1.1 |  | Pordenone | 82 | 0.9 |
| Casarano | 67 | 0.7 |  | Ravenna | 38 | 0.4 |
| Catania | 270 | 3.0 |  | Ridi Abruzzo | 9 | 0.1 |
| Catanzaro | 60 | 0.7 |  | Rimini | 61 | 0.7 |
| Chieti | 216 | 2.4 |  | Rome Opbg | 544 | 6.0 |
| Crotone | 46 | 0.5 |  | Rome Tor-Vergata | 166 | 1.8 |
| Cuneo | 37 | 0.4 |  | S.Giovanni Rotondo | 17 | 0.2 |
| Florence | 410 | 4.5 |  | Sanremo | 35 | 0.4 |
| Forlì | 27 | 0.3 |  | Sassari | 284 | 3.1 |
| Francavilla al Mare | 28 | 0.3 |  | Savona | 32 | 0.4 |
| Genova | 195 | 2.2 |  | Tempio Pausania | 9 | 0.1 |
| Lanusei | 30 | 0.3 |  | Torino | 286 | 3.2 |
| Locri | 42 | 0.5 |  | Tradate | 46 | 0.5 |
| Marche | 246 | 2.7 |  | Trentino | 143 | 1.6 |
| Massa Carrara | 34 | 0.4 |  | Trieste | 47 | 0.5 |
| Messina | 187 | 2.1 |  | Udine | 70 | 0.8 |
| Milan H. Sacco | 98 | 1.1 |  | Varese | 104 | 1.2 |
| Milan S. Raffaele | 535 | 5.9 |  | Verona | 244 | 2.7 |
| Modena | 140 | 1.5 |  | Viterbo | 48 | 0.5 |
| Naples - Federico II | 320 | 3.5 |  | **Total** | **9040** | **100.0** |
